# Endogenous Synthesis of Tetrahydroisoquinoline Derivatives from Dietary Factors: Neurotoxicity Assessment on a 3D Neurosphere Culture

**DOI:** 10.3390/molecules27217443

**Published:** 2022-11-02

**Authors:** Rania Aro, Amandine Nachtergael, Claudio Palmieri, Laurence Ris, Pierre Duez

**Affiliations:** 1 Unit of Therapeutic Chemistry and Pharmacognosy, University of Mons (UMONS), 7000 Mons, Belgium; 2 Department of Neuroscience, Research Institute for Biosciences, University of Mons (UMONS), 7000 Mons, Belgium

**Keywords:** 3D neurosphere, neurodegenerative diseases, essential tremor ET, THIQs, toxicity, Maillard reaction products (MRPs)

## Abstract

Tetrahydroisoquinoline (THIQ) alkaloids and their derivatives have a structural similarity to 1-methyl-4-phenyl-1,2,3,6-tetrahydropyridine (MPTP), a well-known neurotoxin. THIQs seem to present a broad range of actions in the brain, critically dependent on their catechol moieties and metabolism. These properties make it reasonable to assume that an acute or chronic exposure to some THIQs might lead to neurodegenerative diseases including essential tremor (ET). We developed a method to search for precursor carbonyl compounds produced during the Maillard reaction in overcooked meats to study their reactivity with endogenous amines and identify the reaction products. Then, we predicted in silico their pharmacokinetic and toxicological properties toward the central nervous system. Finally, their possible neurological effects on a novel in vitro 3D neurosphere model were assessed. The obtained data indicate that meat is an alkaloid precursor, and we identified the alkaloid 1-benzyl-1,2,3,4-tetrahydroisoquinoline-6,7-diol (1-benz-6,7-diol THIQ) as the condensation product of phenylacetaldehyde with dopamine; in silico study of 1-benz-6,7-diol-THIQ reveals modulation of dopamine receptor D1 and D2; and in vitro study of 1-benz-6,7-diol-THIQ for cytotoxicity and oxidative stress induction does not show any difference after 24 h contact for all tested concentrations. To conclude, our in vitro data do not support an eventual neurotoxic effect for 1-benz-6,7-diol-THIQ.

## 1. Introduction

Dietary factors have been investigated as possible contributing factors in Parkinson’s disease and many other neurological disorders [1]. However, so far, the dietary epidemiology of essential tremor (ET), one of the most prevalent neurological diseases, has not been rigorously studied; however, elevated meat consumption has been reported as a negative effect on ET cases related only to men [2]. Cooked protein foods, such as meat, are a major dietary source for heterocyclic amines (HCAs) and carbonyl compounds which have been linked to neurodegenerative diseases, including essential tremor [3,4,5]. 

**Figure 1:**
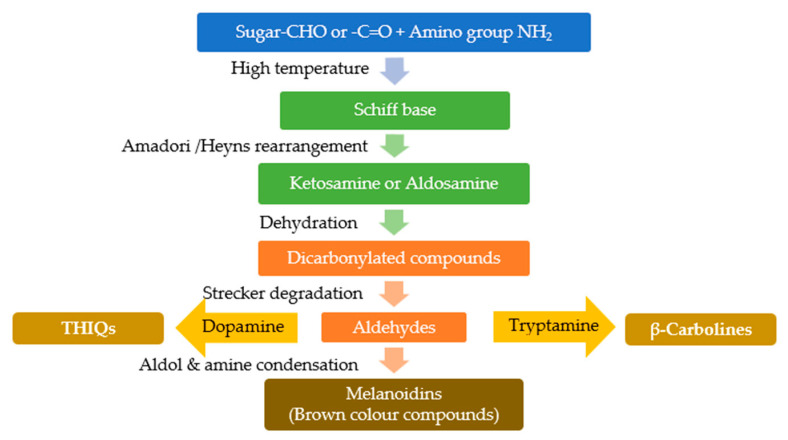
Suggested pathway for the formation of β-carbolines and THIQs through the products of Maillard reaction, as a result of condensation of aldehydes product of Strecker degradation with dopamine or tryptamine.

The Maillard reaction [6] known as the major “cooking reaction”, is a succession of non-enzymatic glycation thermal reactions that provide the basis for the browning colors and aromas characteristic of cooked foods, e.g., grilled meat, roasted coffee, fried onions, or toasted bread. There is a major interest in this reaction for food processing, pharmacology, toxicology, and life sciences [7,8]. The final stage in this reaction includes the aldol condensation of the aldehydes, these reactions are catalyzed by amines to give aldols and N-free polymers, and complex aldehyde-amine polycondensations. The condensation might be with amines such as tryptamine or dopamine, followed by an eventual Pictet-Spengler type reaction, leading to the formation of various β-carbolines (βCAs) and Tetrahydroisoquinolines (THIQs) heterocycles (Figure 1). The end products of Maillard reaction (MRPs) or its intermediary antinutritive compounds are suspected of toxicological effects in animal models, and have been implicated in diseases, notably in neurodegenerative disorders [9,10]. Their quantity in cooked meats varies according to meat type and cooking practices, i.e., temperature, duration, the presence of water, and the concentration of sugar and amino acids, while their identity depends on the reacted sugars and amino acids [11]. For that, factors derived from diet, precisely overcooked meats, and the identification of Maillard reaction products (MRPs) drawn our attention for their possible implication in ET. Even though the Maillard reaction has been widely studied, many of its products are not fully characterized and their biological effects need more investigation. 

**Figure 2:**
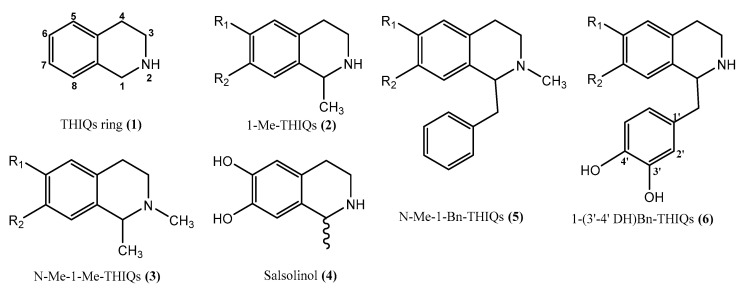
Tetrahydroisoquinolines ring and some derivatives, (**1**) Tetrahydroisoquinolines ring (**2**) 1-methyl-1, 2, 3, 4-tetrahydroisoquinolines, (**3**) 1- methyl-2-methyl-1,2,3,4-tetrahydroisoquinolines, (**4**) salsolinol (1-methyl-6,7-dihydroxy-1,2,3,4-tetrahydroisoquinoline), (**5**) 1-benzyl-1, 2, 3, 4-tetrahydroisoquinolines, (**6**) 1-(3′-4′ dihydroxy)-benzyl-1,2,3,4-tetrahydroisoquinolines.

Tetrahydroisoquinolines (THIQs) are widely distributed among plants, foods, and in the environment; some of them are present in the mammalian brain and, since 1980, they are considered as endogenous compounds. The exogenous compounds have the ability to cross the blood–brain barrier (BBB) [12]. The THIQ family contains different subgroups based on the eventual additional rings connected to the basic structure (Figure 2). The administration of THIQs to a wide range of animal models (primates and mice) was found to reduce the tyrosine hydroxylase (TH) activity, the enzyme responsible for converting tyrosine to dopamine, and dopamine levels in the brain [13], knowing that this reduction implicated in different nervous system diseases. However, not all THIQs have toxic effects, some THIQs in fact increase the levels of glutathione (GSH), nitric oxide (NO), and S -nitrosothiols, with a positive impact on the redox capacities, consequently affording a protection against oxidative stress [14]. So, while some THIQs are potent neurotoxins, such as tetrahydropapaveroline THP (1-[(3,4-dihydroxyphenyl)methyl]-1,2,3,4-tetrahydroisoquinoline-6,7-diol) and 1-benzyl-1,2,3,4-tetrahydroisoquinoline (1BnTHIQ), others, such as higenamine (1-[(4-hydroxyphenyl)methyl]-1,2,3,4-tetrahydroisoquinoline-6,7-diol) and 1-methyl-1,2,3,4-tetrahydroisoquinoline (1-MeTHIQ), are neuroprotectants and have neurorestorative actions [15].

The nervous system has many cell types with multiple functions, complex anatomy, and unique structural and functional characteristics; this complex organization makes the evaluation of neurotoxicity and neuroprotection following an exposure to chemical, biological, or physical influences, a real concern in neurosciences. Neurotoxicity or neuroprotection studies are ideally based on animals; but such in vivo research is becoming less and less resorted to, due to 3Rs regulations (replacing, reducing, and refining) implemented to reduce the number and suffering of research animals. In vivo cells grow within a complex three-dimensional (3D) microenvironment which limits the relevance of 2D in vitro culture systems to an in vivo situation, and makes them the less biologically relevant models, missing complex cell-to-cell interactions [16,17,18,19]. The adjunct of a third dimension to the culture system allows to introduce these important aspects of extracellular matrix, cell-to-cell exchanges, spatial organization, nutrient access, cell geometry, and cell mechanics; those affect various receptors engaged in interactions, modulating cell response and gene/protein expression, closely mimicking the cellular microenvironment [20,21]. Thus, a 3D in vitro model is expected to better mimic the in vivo behavior of cells than 2D cultures [22,23].

A major challenge resides in the increasing number of chemicals to which humans, plants, or the environment are exposed, with possible harmful effects, which underlines the importance to study their eventual toxicity. Therefore, original methods are being devised as “in silico toxicology”, applying statistics and computer science for toxicity assessment or prediction. 

In the present work, we aim to explore the eventual implication of dietary compounds in ET, by investigating carbonyl compounds produced during the Maillard reaction in overcooked chicken that could be implicated in the generation of biologically active artifactual endogenous alkaloids and identify their possible implication in the pathogenesis ET on an original 3D neurosphere model.

## 2. Results

### 2.1. Identification of Carbonyl Compounds in Overcooked Chicken Meat

#### 2.1.1. Derivatization with 4-hydrazino-N, N, N-trimethyl-4-oxobutanaminium Iodide (HTMOB) and Analysis by ESI-MS/MS

**Figure 3:**
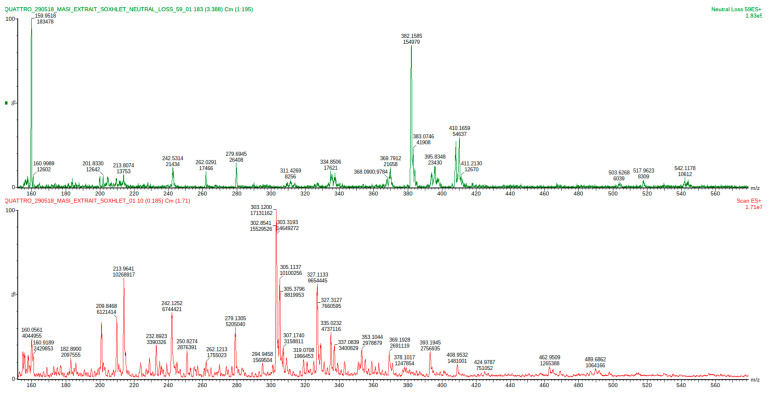
Mass spectrum of the HTMOB-derivatized carbonyl extract obtained from overcooked chicken meat by condensation with silica-gel supported reagent. ESI-MS full scan mode (below) and ESI-MS/MS neutral loss 59 Da mode (above).

Based on this method, the carbonyl extracts obtained from overcooked meat chicken and the browned meat juice by condensation with silica-gel supported reagent were analyzed. The process of identifying the carbonyl compounds consisted in defining the precursor molecular ion *M+* corresponding to a peak in the neutral loss scan (59 Da), then examining the fragmentation patterns that allowed to deduce the identities of peaks, as described by Johnson DW (2007) [24]. The comparison of spectrum with created database for 12 reference aldehydes, selected for their attested presence in cooked meats [25]; allowed to identify: glycolaldehyde, isobutyraldehyde, isovaleraldehyde or 2-methylbutyraldehyde (the same mass) and phenylacetaldehyde in overcooked chicken extract (Figure 3, Table 1).

**Figure 4:**
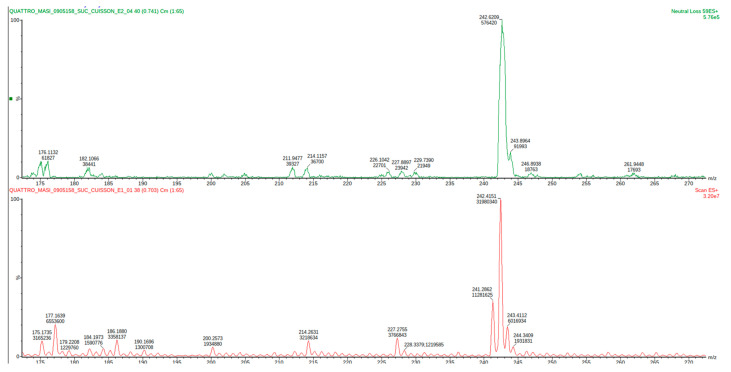
Mass spectrum of the HTMOB-derivatized carbonyl extract obtained from browned meat juices by condensation with silica-gel supported reagent. ESI-MS full scan mode (below) and ESI-MS/MS neutral loss 59 Da mode (above).

The same operation was repeated with the browned meat juices extract and these aldehydes could be identified: isobutyraldehyde, isovaleraldehyde or 2-methylbutyraldehyde (the same mass), acetaldehyde, and formaldehyde (Figure 4, Table 2).

These analyses were repeated three times (technical replicates), yielding sensibly similar spectra that allowed to identify the same compounds. 

#### 2.1.2. Analysis of Volatile Carbonyl Compounds by Derivatization with PFBHA Followed by Gas Chromatography-Mass Spectrometry (GC/MS)

**Figure 5:**
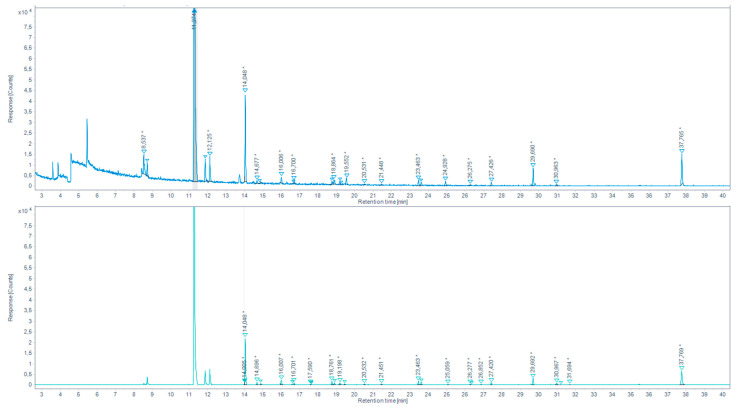
Total ion chromatogram (TIC) for the browned chicken meat juices extracted by SAFE and derivatized with PFBHA (up) and the extracted-ion chromatogram (EIC) 180.7–181.7 *m/z* (down) (* indicates that the peak has been manually integrated).

**Figure 6:**
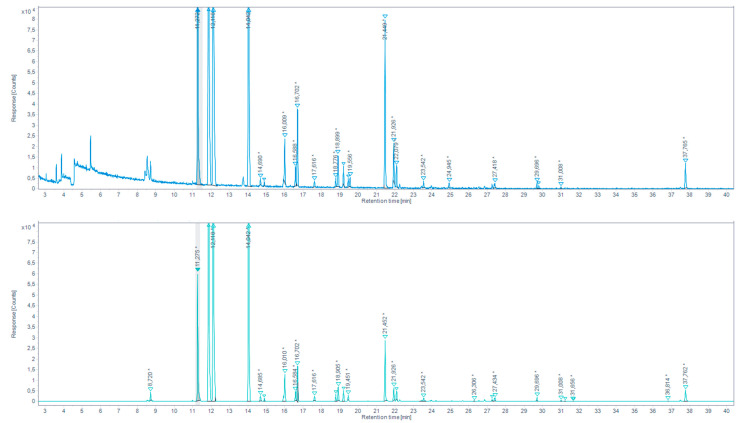
Total ion chromatogram (TIC) for the aqueous chicken sample extracted by SAFE and derivatized with PFBHA (up) and the extracted-ion chromatogram (EIC) 180.7–181.7 *m/z* (down) (* indicates that the peak has been manually integrated).

The derivatization with PFBHA was applied following two different methods:

1. EPA method 556 on the SAFE extract:

A reference table of retention times (RT), Kovats retention indices (KRI) and mass spectra was built, in our GC-MS condition, based on SAFE extract realized on *“Aldehydes mixtures-556”*. In total ion chromatograms (TIC), the peaks corresponding to these reference standards were identified by their RT and KRI, according to EPA method 556, their C6F5 CH2 fragment (characterized through an extracted-ion chromatogram (EIC) *m/z* 180.7–181.7), and a mass spectrum match in the NIST 2017 database. Carbonyl analytes were tentatively identified by the same characteristics, comparing the RTs, KRIs, and fragmentations of reference peaks with those in the chromatograms of the SAFE distillates obtained from aqueous extracts of browned chicken meat juice (Figure 5, Table 3), and overcooked chicken meat (Figure 6, Table 4) a blank injection allowed to identify eventual procedural oximes as recommended by EPA method 556. For any sample peak RT that was not corresponding to a reference peak, the MS spectrum was searched through the NIST database. 

We were able to identify a series of aldehydes in both browned chicken meat juice and overcooked chicken meat aqueous extract SAFE distillates. For the continuation of the work, a series of aldehydes presenting the higher response peaks and related to the standard references and/or yielding a high prediction match in the NIST database were selected. These are: propionaldehyde, isobutyraldehyde, hexanaldehyde, benzaldehyde.

2. Direct derivatization by headspace single-drop microextraction (HS-SDME) with droplet PFBHA derivatization

AS in the previous method, we built a reference table of retention times (RT), Kovats retention indices (KRI) and mass spectra based on a derivatization in the PFBHA hexane droplet on the aqueous solution of the “Aldehydes mixtures-556, in our derivatization and GC-MS conditions. The identification of analytes in total ion chromatograms (TIC) was performed based on the mix standard created database, and after the exclusion of peaks present in the procedural blank. Whenever a samples peak RT was not corresponding with any reference peaks, its MS spectrum was searched through the NIST database. Note that, as seen with the standard aldehydes mix, the suggested identification match probability seriously decreases with peak size.

**Figure 7:**
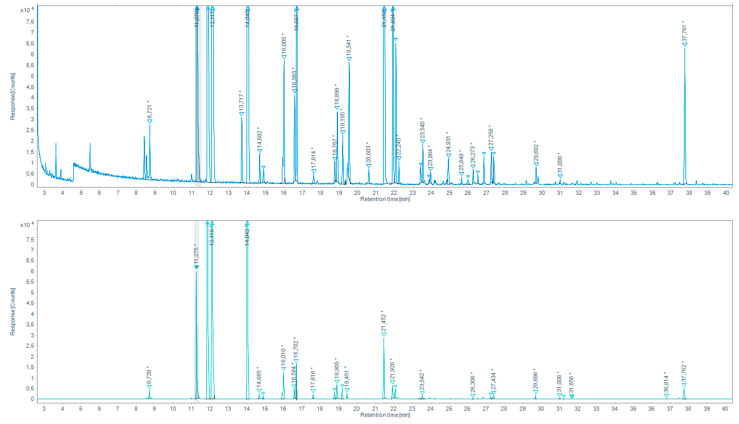
Total ion chromatogram (TIC) for browned chicken meat juices extracted by HS-SDME on PFBHA droplet (up) and the extracted-ion chromatogram (EIC) 180.7–181.7 EI (down). (* indicates that the peak has been manually integrated).

**Figure 8:**
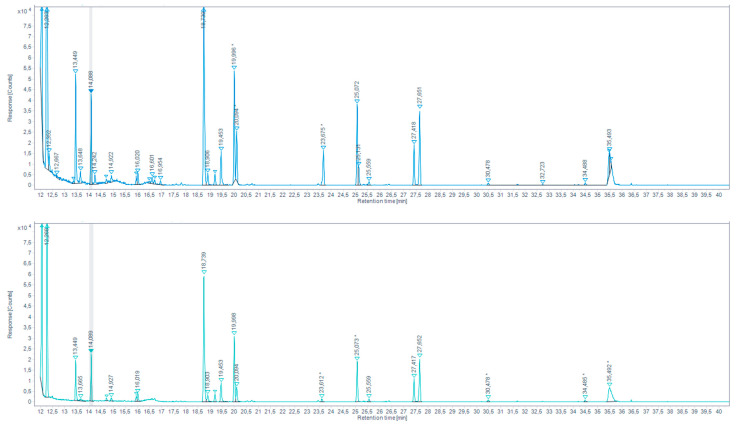
Total ion chromatogram (TIC) for aqueous chicken meat extract sample extracted by HS-SDME on PFBHA droplet (up) and the extracted-ion chromatogram (EIC) 180.7–181.7 EI (down) (* indicates that the peak has been manually integrated).

With the microdroplet method, we were able to identify different carbonyl compounds in the browned chicken meat juice (Figure 7, Table 5) and in overcooked chicken meat aqueous extract (Figure 8, Table 6). For the continuation of the work, the aldehydes with the higher response peaks were selected. For the browned chicken meat juice: propionaldehyde, butyraldehyde, hexanaldehyde, benzaldehyde. For the overcooked chicken meat aqueous extract: propionaldehyde, hexanaldeyde.

### 2.2. Identification of Carbonyl Compounds Likely to React with Endogenous Amines to Form “Artifactual” Alkaloids

#### 2.2.1. Identification of Artifact by TLC

A series of carbonyl compounds reference standards, from the compounds detected in the different extraction method, were investigated for a possible condensation with biogenic amines (dopamine, serotonin, GABA, nor-epinephrine, tryptamine, and tryptophane). The verification of reaction possibility was performed using TLC techniques that is a semi-specific method of analysis; despite its low-resolution, it has a distinct advantage of allowing post-chromatographic in situ reactions and is frequently used as a preliminary screening technique, offering valuable data regarding the identity of the components present in a given sample. The results obtained by TLC should however be confirmed by other analytical methods. The occurrence of a reaction was confirmed on TLC plates, by observing the appearance of a new spot that is not present in either of the reactants and/or the disappearance of a reactant spot. As a result, 12 reactions were identified and listed in Table 7.

#### 2.2.2. Identification of Reaction Products by HPTLC/MS 

The verification of reaction possibility was performed using TLC techniques and the reaction products were tentatively identified by TLC/MS. It should be noted that TLC/MS is unable to precisely show whether a cyclisation has effectively taken place as the expected molecular weight (*m/z*) would be the same for cyclic and non-cyclic derivates; thus, further verification is needed to confirm the identity of reaction products of interest.

**Figure 9:**
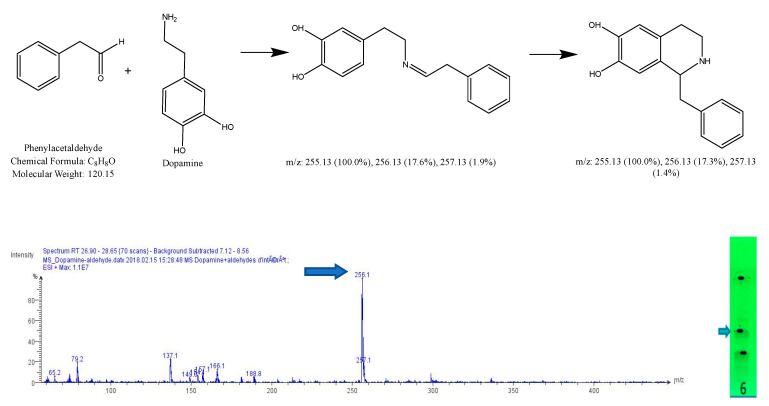
Expected reaction between dopamine and phenylacetaldehyde. Mass spectrum obtained for the 2nd spot of the reaction mixture on MS grade TLC plate.

**Figure 10:**
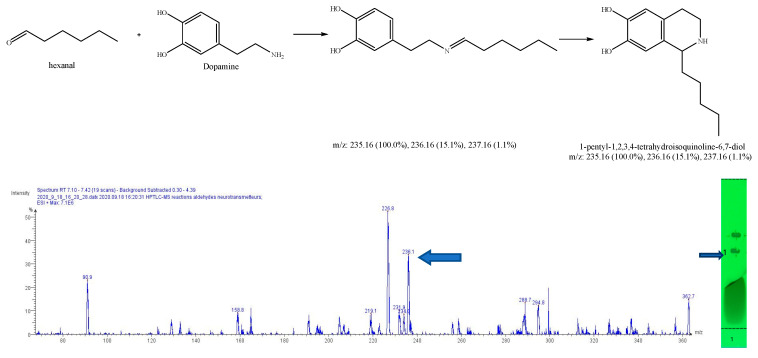
Expected reaction between dopamine and hexanal. Mass spectrum for the 1st spot of the reaction mixture on MS grade TLC plate.

**Figure 11:**
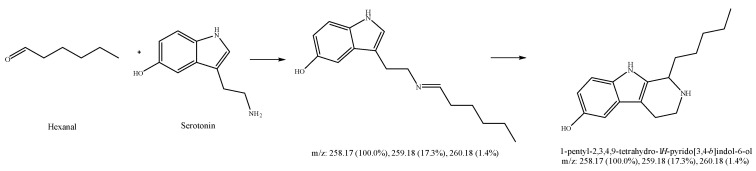

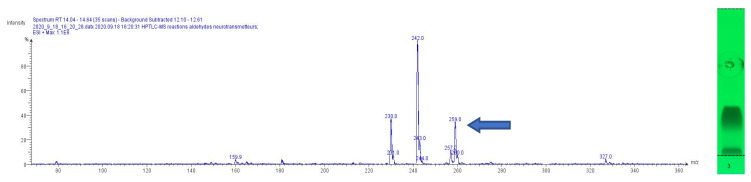
Expected reaction between serotonin and hexanal. Mass spectrum for the spot of the reaction mixture on MS grade TLC plate.

**Figure 12:**
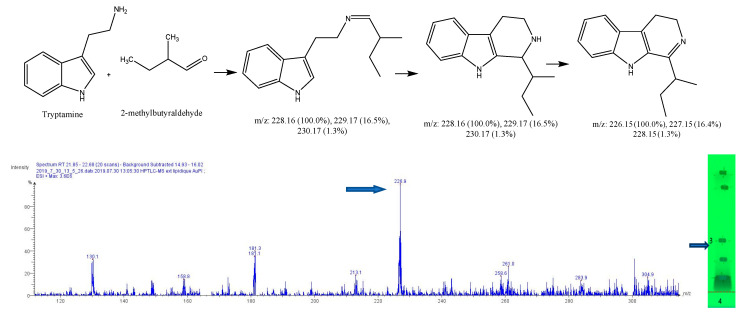
Expected reaction between 2-methylbutyraldehyde and tryptamine. Mass spectrum for the 3rd spot of the reaction mixture on MS grade TLC plate.

**Figure 13:**
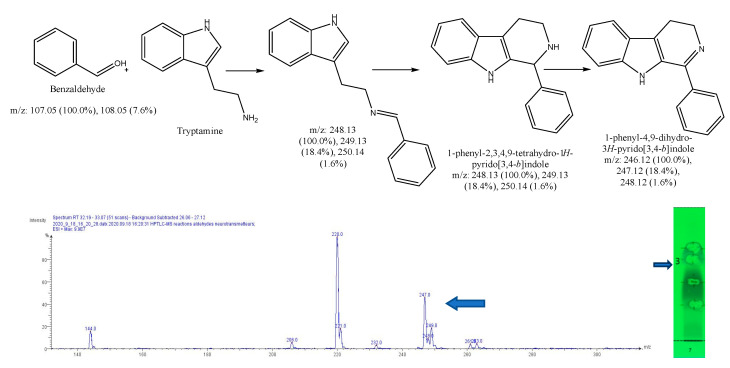
Expected reaction between tryptamine and benzaldehyde. Mass spectrum for the 3rd spot of the reaction mixture on MS grade TLC plate.

**Figure 14:**
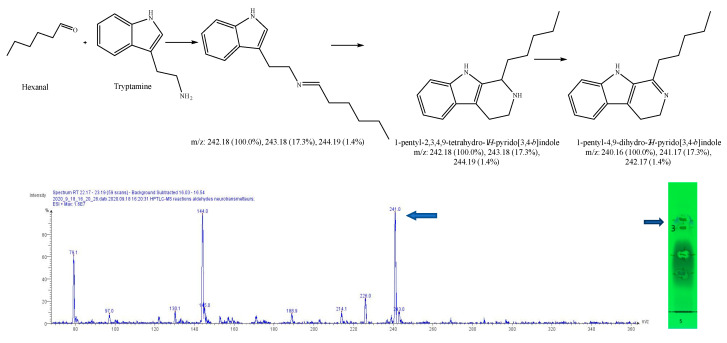
Expected reaction between tryptamine and hexanal. Mass spectrum for 3rd spot of the reaction mixture on MS grade TLC plate.

After subtracting the spectrum of a blank spot dialyzed in the same conditions, a specific mass was researched in the MS spectrum for each reaction, based on forecasted theorical reaction(s), taking into consideration that both the cyclic and acyclic reaction products may present the same mass. As our interest is focused on condensation reactions followed by spontaneous cyclization to produce β-carbolines or THIQ, further identification is then generally required. As a result, 6 products of reaction were identified, with dopamine 1-benz-THIQ- diol (Figure 9), and 1-pentyl-THIQ-diol (Figure 10); with serotonin 1-pentyl-THβCa-ol (Figure 11); and with tryptamine 1-isobutyl DhβCa (Figure 12), 1-phenyl-DhβCa (Figure 13) and 1-pentyl-DhβCa (Figure 14).

### 2.3. In Silico Prediction of Neurotoxicity or Neuroprotection Effect

#### 2.3.1. In Silico Prediction with eMolTox

The neurotoxicity was predicted for six molecules using the publicly available web server e-MolTOx. 

For 1-Benz-THIQ-diol, the e-MolTOx algorithms defined a possible modulation of dopamine D1 receptor that might cause an injury to central nervous system, kidney, and heart (*p* = 0.999 confidence). This is probably an inference from the catechol C6-C2-N structure. 

1-Isobutyl-DhβCa is suggested to modulate adrenergic receptors α-2A and α-2B, in addition to serotonin 2b receptor. The injury affects the CNS and nervous system subsequently with 0.990 confidence.

1-Pentyl-THIQ-diol potentially modulates dopamine D1 and serotonin 1b receptors with 0.990 confidence. This is also probably an inference from the catechol C6-C2-N structure.

1-Pentyl-ThβCa-ol is predicted to modulate dopamine D1 and serotonin 1b receptors with confidence of 0.994 and 0.990.

1-Pentyl-DhβCa is suggested to modulate adrenergic receptors α-2a and α-2b of the nervous system with confidence of 0.990.

Finally, 1-Phenyl-DhβCa has a potential toxicity on nervous system with confidence of 0.980 by modulating the serotonin 2b receptor.

#### 2.3.2. In-Silico Prediction with pkCSM

The pkCSM web server predicts the pharmacokinetic properties (ADMET, i.e., absorption, distribution, metabolism, excretion, and toxicity), that is important to understand a possible activity of molecules, we focused on absorption and distribution in order to pursuit the possibility to reach the receptor in the brain and below are the main prediction outcomes for absorption and distribution:

1-Absorption:

All molecules are not water soluble (negative result for log S), which affects the absorption, but very well absorbed from the human intestine (Caco2 > 0.9 and intestinal absorption > 30%). However, they are likely to be substrates of the P-glycoprotein.

2-Distribution:

All molecules are likely to present a high volume of distribution in tissue vs. plasma (log VDss > 0.45), except 1-Phenyl-DhβCa but it is still not considered as low (log VDss < −0.15). On the other hand, the fraction unbound to serum proteins is lower than the bound one, which negatively affects the cellular membrane diffusion or transport.

Moreover, only 1-phenyl-DhβCa are considered readily to cross the blood brain barrier with log BB > 0.3. Although the CNS permeability factor, that takes in consideration the compound surface area, indicates that all compounds with (log PS > −2) can penetrate CNS; 1-Benz-THIQ-diol and 1-pentyl-THIQ-diol are however less able to penetrate the CNS with (−3 < logPS < −2).

#### 2.3.3. In-Silico Screening with PubChem

The search for 1-Benz-THIQ-diol in the section “biological test results” demonstrates that the molecule presents a binding affinity toward D2 and D1 dopamine receptors but at different concentrations, as shown in rat striatal synaptosomes [26]. This affinity could explain a D1, D2 dopaminergic antagonist effect [27]. Knowing that dopamine antagonist agents are known to induce parkinsonism, dystonia, tics, and tremor [28,29], accordingly the compound was selected for further investigations.

1-Pentyl-THIQ-diol- presents in the “literature” section one article investigating the antimicrobial, antimalarial, cytotoxic, and anti-HIV potentiality of isoquinoline and benzylisoquinoline alkaloids [30].

The search of 1-Phenyl-DhβCa demonstrates in “biological test results” section an antimicrobial activity [31].

1-Isobutyl-DhβCa, 1-Pentyl-6-ol-ThβCa, and 1-Pentyl-DHβCa do not exist in the PubChem database.

### 2.4. In Vitro Evaluation of Neurotoxicity and Neuroprotection on 3D Neurosphere

#### 2.4.1. Evaluation of Cell Viability

**Figure 15:**
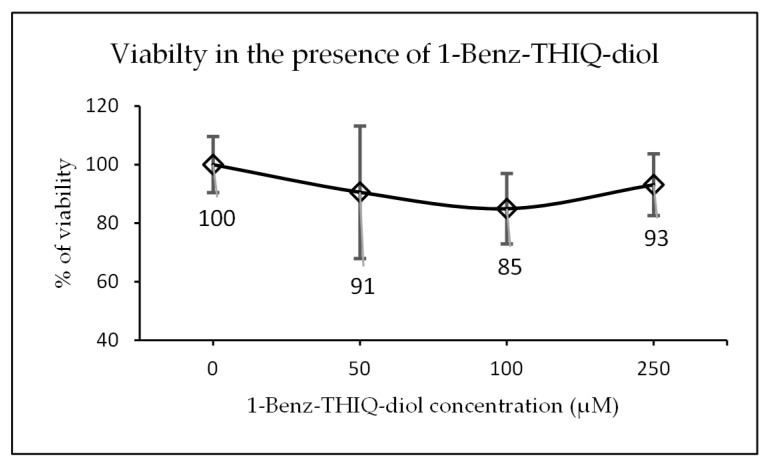
Viability after 24 h exposure to different concentrations of 1-Benz-THIQ-diol, data from four biologically independent experiments performed with three technical replicates (resazurin viability test; mean ± SD) (N = 12). There is no significant difference between concentrations (ANOVA; *p* = 0.150).

After exposure of 3D neurosphere at day 14 of culture to increasing concentrations of 1-Benz-THIQ-diol (50, 100, 250 µM) for 24 h, the 3D neural spheroid showed a slight decrease in viability (resazurin test), which was not 1-Benz-THIQ-diol concentration-dependent. The mean values of percentage of viability based on control group were 90.6 ± 22.6 (SD), 84.9 ± 12.1, and 93.1 ± 12.6% for 1-Benz-THIQ-diol 50, 100, and 250 µM, respectively (Figure 15). A two-way ANOVA analysis for factors “treatment”, “experience”, and “treatment × experience” indicates there is no statistically significant difference among the different treatments (*p* = 0.150), the different experiences (*p* = 0.051), and no interaction experience-treatment (*p* = 0.880).

#### 2.4.2. Reactive Oxygen Species (ROS) Measurement

**Figure 16:**
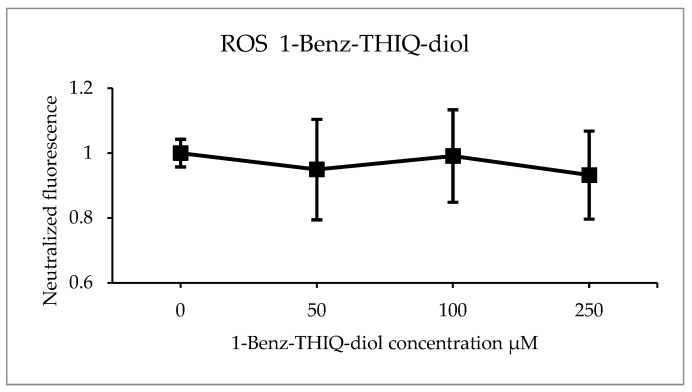
ROS production (percentage of control) after 24 h exposure to different concentrations of 1-Benz-THIQ-diol, data from five biologically independent experiments performed with three technical replicates (mean ± SD) (N = 15). There was no significant difference between the treatments (two-way ANOVA, *p* = 0.115).

The DCFH test was applied after 24 h of exposure to 1-Benz-THIQ-diol, on five biologically independent experiments performed with three technical replicates (Figure 16). After analyzing the fluorescence intensity, we found that there is no significant effect of 1-Benz-THIQ-diol on the generation of ROS as measured here (two-way ANOVA, *p* = 0.348 factor “Treatment”; *p* = 0.391 for the interaction “Culture” × “Treatment”), while the difference was statistically significant for factor “Culture” (*p* = 0.013) and the pairwise comparisons (Bonferroni t-test) indicate that the difference was significant between C1 and C2 (*p* = 0.014).

## 3. Discussion

ET is a widespread neurodegenerative disease whose etiology is still unclear, while both genetic and environmental factors are suspected. Many findings support the heterogeneity of essential tremor pathology [32,33], which makes it challenging to define the role of a specific neurotoxin or a particular factor in its etiology. Harmane, a β-carboline alkaloid has been incriminated in the pathology of ET [34,35], and overcooked meats, as a resource for harmane (3.8–8.5 ng/g [11,36]), has been implicated. We however raise some doubts on the alleged link between overcooked meat, β-carbolines, and essential tremor; it is noticeable that this link, pledged for by different authors [36,37,38,39], is attributed to “*lifelong exposure with brain accumulation of these highly lipophilic compounds*”. Our doubts are comforted by the lack of link between essential tremor and tobacco smoking (tobacco smoke contains β-carbolines; 1 cigarette corresponds to 207–2780 ng harmane [40]) or using the medicinal plant *Peganum harmala L.* (rich in β-carboline alkaloids; the seeds contain harmaline, harmine, harmalol, harmane and harmol at 3.8, 2.93, 0.12, 0.03 and 0.02%, respectively [41]). Given the difficulties and the dubious interest in measuring such low amounts of β-carbolines in meat, we propose a methodology for investigating overcooked meats for compounds that could be implicated in the generation of biologically active artifactual endogenous alkaloids”.

Carbonyl compounds have special interest for their toxicity and carcinogenicity that relate to their ability to adduct or react with endogenous amines, proteins, or nucleic bases [42]. Many studies examined their (trace) presence in air, water, beverage, or food; as such, different sensitive methods were developed to identify and quantify nanogram amounts of carbonyl compounds, making profit of various derivatization reagents capable to capture small (a) polar carbonyl molecules [43,44,45,46]. Carbonyl compounds play a significant role in the meat flavor, also they are very reactive toward oxidoreductive, polymerization, and amine-condensation transformations and represent intermediates in a lot of biochemical reactions. Our work aimed at investigating the dietary epidemiology of essential tremor, through the study of overcooked meats and the development of a strategy to identify Maillard reaction products (MRPs) possibly implicated in ET. Our interest was focused on the possibility of aldol condensation of carbonyl compounds with amino groups of meat constituents or endogenous amines to produce heterocyclic amines (i.e., artifactual and endogenous alkaloids, respectively). The production of carbonyl compounds in heated meat might be a result of Maillard reaction, Strecker reaction (that is perceived as a reaction within the Maillard reaction), lipid oxidation, degradation of thiamine, or even bacterial action [25,47]. 

The preparation of our samples aimed at developing a brown color, that results from the condensation of amino compounds and sugar fragments in the meat into brown pigments called melanoidins, as indicator of Maillard reaction occurrence. By contrast with some studies in the literature, we have chosen to develop an overcooking method that yields meat remaining consumable, typical of what can be achieved in daily (over)cooking.

For carbonyl compounds extraction, we used different methods to extract the volatile and non-volatile carbonyls from overcooked chicken meat. For volatile carbonyls, the SAFE method was described as one of the best methods for “clean” aroma extracts at low temperature, with high efficiency of extraction for the most volatiles compounds [48]; the amount of solvent used in this method is minimal and the complexity of the work is considered low. The HS-SDME technique that allowed the isolation of numerous volatile carbonyl compounds with small sample volume and very few steps, but needs precision in application and definition of the best parameters for the samples, this technique is fast, inexpensive, and ensures reliable results [49,50].

For non-volatile carbonyls, we relied on a Soxhlet apparatus for extraction then condensation with a semicarbazide for the isolation of lower-polarity carbonyl compounds from other extracted impurities. The method was adequate but is a long procedure, requiring huge volumes of solvent, is time consuming, and absolutely not adequate to recover volatile aldehydes [51].

Concerning the identification, we applied different derivatization strategies and chromatographic methods. Conducive to volatile carbonyls the derivatization with PFBHA was applied, following two different methods: (i) the EPA method 556 [52] on the SAFE extract; and (ii) a direct derivatization by headspace single-drop microextraction (HS-SDME) [49]. Comparison of these two analytical methods indicates a particularly good agreement between the two methods for the identification of volatile aldehydes. The SAFE technique allows a clearer chromatogram (better signal to noise ratio); however, HS-SDME is way faster and requires much less steps.

Toward the non-volatile carbonyls, the identification was aided by derivatization with HTMOB, a modified Girard derivatizing reagent. This HTMOB derivatization is a universal ESI-MS/MS product ion, with a constant mass loss regardless of molecule size, yielding a high intensity signal, which improves the analytical sensitivity [24]. The ESI-MS-MS analysis of HTMOB-reacted overcooked chicken meat carbonyl extract (silica gel extract) provided a rapid and clean neutral loss 59 spectrum but indicated the need to create a database of carbonyl standards to aid in peaks identification.

We examined the possibility of condensation reactions with six endogenous amines followed by spontaneous cyclization to produce an isoquinoline ring (reaction with dopamine, epinephrine, or norepinephrine) or a β-carboline ring (reaction with serotonin or tryptamine). The verification of reaction possibility was performed using TLC techniques and the reaction products were tentatively identified by TLC/MS). As a result, 4 of the tested carbonyl compounds were demonstrated to be able to spontaneously condensate with amines, with six reaction products yielding the *m/z* corresponding to expected reaction products. As TLC/MS cannot give us information about the nature of the molecule (aliphatic or cyclic), further verification was applied for the molecules of interest.

The practice of “in silico toxicology” allows to predict potential risks for molecules, in a cost effective and timely manner. Different methodologies can be used as preliminary screening tools to identify potential toxicant effect and prioritize compounds for further testing by standard laboratory procedures [53,54]. It is beneficial to use the results of several expert toxicity systems to reduce the risks of false negative predictions. In this work, the neurotoxicity was predicted for 7 molecules using the publicly available web server e-MolTOx and the results were crosslinked with the biological activities gathered from PubChem. In addition, the pharmacokinetic properties (ADMET) were predicted in silico, using the pkCSM web server.

According to the in silico predicted toxicity of reaction products, the experimental work was continued with the major reaction product between dopamine and phenylacetaldehyde, i.e., the 1-benzyl-1,2,3,4-tetrahydroisoquinoline-6,7-diol (1-benz-6,7-diol THIQ). Our trials to purify enough 1-benz-6,7-diol THIQ for NMR analysis were unsuccessful, the yield of synthesis being quite low and the purification particularly difficult due to an apparent reversibility of the condensation reaction. A commercial reference was then resorted to for comparing TLC Rfs and MS to confirm that 1-benz-6,7-diol THIQ is effectively a major product of reaction when incubating dopamine with phenylacetaldehyde in physiological conditions. The compound has a good probability to cross the blood–brain barrier and to encounter dopamine in brain tissues. Moreover, given the very high concentrations of dopamine in the gastrointestinal tract (production by enteric neurons and intestinal epithelial cells), and thus in hepatic portal vein [55], there is also a high probability of local condensation with alimentary phenylacetaldehyde to yield 1-benz-6,7-diol THIQ. The predicted and reported affinity with dopamine receptors could explain a D1, D2 dopaminergic antagonist effect [27]. Considering the compound predicted pharmacokinetic properties (pkCSM: log P, 2.527; log BB, 0.109; log PS, -2.053) and knowing that dopamine antagonists are known to induce parkinsonism, dystonia, tics, and tremor [28,29], the compound was accordingly selected for further investigations.

In the interest of neurotoxicity examination, we have chosen 3D neurosphere since the 3D cell cultures and spheroids are being increasingly used to bridge the gap between in vitro 2D cell cultures and in vivo animal models. There is a consensus that 3D models are more physiologically relevant than 2D cell cultures and biochemical assays, as they more closely represent the microenvironments, cell-to-cell interactions, and biological processes that occur in vivo [56,57]. 3D neural spheroids represent a promising future in research for neurological diseases as they will allow to conduct screening studies to identify toxicological effects of environmental factors. In addition, the primary location of the CNS sites involved in the generation of ET remains highly debated. It is possible to select brain structures during cell harvesting for seeding and so to produce organoids specific to parts of the brain, possibly leading to specific architectural and/or functional models. This theoretical prospect would permit the study of THIQs in cells from the cerebellum, cortex, or brainstem, possibly providing additional evidence to support the key-role of one target rather than another. The 3D neural spheroid model presented in this work has shown its ability to yield and reproduce 3D culture, using simple materials and routine laboratory equipment. Despite the challenges in 3D culture and data obtaining, this 3D neural spheroid model mimics a neuronal microenvironment, allowing a fine study of neurodegenerative disorders and the effect of chemicals on the brain.

For cytotoxicity assays, we have assessed 1-benz-6,7-diol THIQ at different concentrations, over 24 h. The application of the resazurin test on spheroids exposed to different concentration of 1-benz-6,7-diol THIQ does not indicate a statistically significant reduction in viability. This result confirmed previous result that demonstrated the relation of neurotoxicity effect of 1-benz-6,7-diol THIQ with cell types, more specifically the neurotoxic effect is related to dopaminergic neurons [58].

In the interest of ROS generated within cells, which could also be reflected by ROS leakages into the culture medium [59,60], 2′,7′- dichlorodihydrofluorescein (DCFH) assay was applied for the analysis of extracellular ROS. The evaluation of leaked ROS after 24 h contact does not show any difference for all tested concentrations of 1-benz-6,7-diol THIQ.

However, ET is a neurodegenerative disease that progresses with time, and our research was limited to the study of short-term effects of molecules on 3D spheroids. For better understanding the implications of these molecules in ET, future studies should address mid- to +/− long-term effects of the molecules on the spheroids, for example by regular addition of test molecules to the culture media along spheroid life; to study effects on brain development (a possible mimic of plasticity?), exposure may possibly start during spheroid growth, maybe even starting on culture day 1.

## 4. Materials and Methods

### 4.1. Preparation of Overcooked Meat Samples

The preparation of our samples aimed at developing a brown color as indicator of Maillard reaction occurrence. By contrast with some studies in the literature, we have chosen to develop an overcooking method that yields meat remaining consumable, typical of what can be achieved in daily (over)cooking.

The breast chicken was purchased from a local supermarket and was cut to very thin slices and was broiled in a domestic oven at 200 °C for 40 min, the slices were turned to have a homogenous brown color on the two sides. Then it was ground by a powerful ball mill (RETSCH MM400), the vibration frequency was 300 min^−1^ for 30 s. The meat fond (browned chicken juices) was collected from the oil-less overcooking of 1000 g chicken with 250 mL distilled water and filtered with a filter paper. The aqueous sample of overcooked chicken was extracted with deionized water in a 1:5 ratios (*w/v*), in a closed vessel placed in an ultrasonic bath (30 °C for 6 h), then centrifuged (10 min, 2000× *g*) to collect the supernatant.

### 4.2. Extraction of Overcooked Samples

#### 4.2.1. Extraction of Volatile Carbonyl Compounds by Solvent Assisted Flavor Evaporation (SAFE)

To extract volatile compounds, we applied a method designed for the isolation of volatile molecules from non-volatiles in solvent extracts, relying on an innovative distillation unit (a SAFE unit); the principle is based on high-vacuum transfer, which ensures a fast and careful isolation of volatiles from complex matrices [61]. For this method 22 mL of aqueous samples, meat fond, deionized water (procedural blank), and “Aldehydes mixtures-556”, diluted at 40 ng/mL in deionized water were extracted in triplicate, and the SAFE apparatus was cleaned by distillation of 10 mL of water between the samples.

#### 4.2.2. Extraction and Isolation of Volatile Carbonyl Compounds by Headspace Single-drop Microextraction (HS-SDME) with Droplet Derivatization

The microextraction, an analytical technique, was first described by Jeannot, M.A. (1996) [50]. This method was modified to head-space for droplet derivatization with O-(2,3,4,5,6-pentafluorobenzyl) hydroxylamine hydrochloride (PFBHA) by Li et al. (2005) [49]. The head-space extraction is a dynamic gas phase extraction to quantify volatiles in solid or complex liquid samples, and derivatization of aldehydes with PFBHA to form the corresponding oximes is very well-known, rapid, and a complete reaction. Several parameters can affect HS-SDME with droplet derivatization: (i) The choice of the extraction solvent that depends on its low volatility, its ability to extract the analytes, and its peaks that should be well separated from the analyte peaks in the chromatogram; (ii) the temperature of extraction which should be high enough to ensure the vaporization of analytes and their absorption in the microdroplet without evaporating the microdroplet solvent (loss of extraction solvent); (iii) the stirring rate that is particularly important to enhance the mass transfer in the aqueous phase, and decrease the time needed to reach the thermodynamic equilibrium between aqueous phase and headspace without affecting the stability of droplet; and (iv) the volume of organic solvent; while using a large organic drop results in an increased analytical response, larger drops are difficult to manipulate and are not reliable. Therefore, heptane was selected for our extraction, the temperature was fixed at 60 °C, the stirring at 450 rpm, the volume of organic solvent at 3 µL, and the time of extraction at 6 min. We applied this method on the same samples of SAFE method.

The solution of PFBHA in heptane was prepared by dissolving 20 mg of PFBHA into 1 mL deionized water; the 1 mL of PFBHA aqueous solution was extracted with 1.0 mL of heptane; the expected final concentration of PFBHA is about 16 mg/mL [49]. For each extract, 1 mL of sample and 10 µL of 0.6 mM methyl ethyl ketone in water (internal standard) were transferred to a closed 4 mL vial (amber screw vial 45 × 14.7 mm, BGB) containing a small magnetic rod. The vial was placed in a water bath at 60 °C under stirring and a drop of 3 µL of PFBHA in heptane was suspended from the micro-syringe.

#### 4.2.3. Extraction and Isolation of Carbonyl Compounds by Condensation with a Silica-gel Supported Reagent

**Figure 17:**
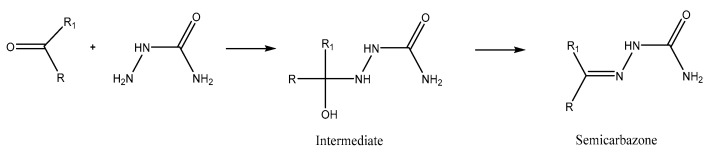
The condensation of a carbonyl group with a semicarbazide group to form a semicarbazone.

The extraction is based on the reactivity of the carbonyl group toward a semicarbazide reagent to form a semicarbazone (Figure 17: The condensation of a carbonyl group with a semicarbazide group to form a semicarbazone). This condensation ensures the separation of carbonyl compounds from other solutes in an organic solvent (hexane or toluene) extract, in which the semicarbazones are invariably insoluble. Singh et al. (1979) [62] further developed the concept by proposing a solid phase reagent that we applied to profile aldehydes in our meat samples.

To prepare of the silica-gel supported reagents, semicarbazide hydrochloride (5.0 g; 0.045 mole) was added to a solution of NaOH (2.0 g; 0.05 mole) in water-MeOH (1: 1; 60 mL). To the resulting clear solution, silica gel (fraction 80–200 mesh, bulk density, 0.71 g/mL; 45 g) was introduced with stirring. Subsequently, the whole mixture was mechanically shaken (1 h) at 30–35 °C and water–MeOH was removed on a rotary evaporator (90 °C; 80–90 mm Hg; 30–45 min) to yield a white free-flowing powder. The final reagent was stored in a brown bottle at room temperature.

In pursuance of extraction and condensation of ketonic/aldehydic material, a meat sample (165 g) was transferred in a Soxhlet apparatus and extracted with toluene (350 mL) for 18 h. After concentration to about 100 mL, the toluene extract was added with 30 g of the semicarbazide-silica reagent. The mixture was heated (70 ± 2 °C) and stirred for 12–18 h, the absence of the carbonyl compound in the solution was determined at the end of this step, through the absence of yellow to orange spots in a TLC test when spraying a 0.005 M 2,4-dinitrophenylhydrazine (DNPH) solution in ethanol. Next, the mixture was cooled, filtered, and the solid was washed with the same solvent (4 × 50 mL, room t°); the absence of aldehydes/ketones was similarly verified in the wash solvent. The solid phase containing the semi-carbazones was added to a solution of oxalic acid (9 g) in water (160 mL); after addition of toluene (100 mL), the mixture was stirred and refluxed for 4–5 h. After cooling, the liquid phase was transferred to a separatory funnel and the silica gel was washed with toluene (50 mL × 2) and also transferred to the funnel. After the separation of phases, the aqueous phase was extracted with toluene (100 mL × 2) and the extracts were combined with the main toluene extract. The combined toluene extracts were washed with water (100 mL × 2) and with a saturated NaCl solution (~26%; 50 mL), then dried with anhydrous sodium sulfate (Na2SO4). Finally, the solvent was transferred in a weighed balloon, evaporated to dryness, and the regenerated carbonyl compounds were weighed.

This extraction ends up by distillation of toluene to yield the final extract; despite the use of a fractionated distillation (vacuum distillation with a Vigreux column), we could not recover a fraction with a boiling point lower than toluene. This indicates the loss of all volatile carbonyl compounds; therefore, this method was intended for the non-volatile compounds.

### 4.3. Characterization of Extracts

Environmental aldehydes are in general small polar and volatile molecules that are biochemically unstable; for this reason, derivatization with different reagents is widely used in their identification and quantification. Derivatization can also improve volatility, chromatographic separation and peak symmetry, MS ionization, MS/MS fragmentation, and the analytical sensitivity.

In this work the derivatization reagent *O*-2,3,4,5,6-(pentafluorobenzyl)hydroxylamine (PFBHA) was applied to detect the volatile carbonyl compounds, the reaction of PFBHA with carbonyl compounds being described as instantaneous. Analyses were performed by gas chromatography coupled with mass spectrometry (GC/MS) in total ion chromatogram (TIC) and extracted ion chromatogram (EIC) modes. The aldehyde-PFBHA oxime derivatives are clearly identified by the presence of the *m/z* 181 fragment (C_6_F_5_-CH_2_).

The SAFE extraction was followed by derivatization with PFBHA, based on the United States Environmental Protection Agency EPA method of June 1998. The extract was analyzed on a gas chromatograph equipped with a split/splitless injector, and a mass spectrometry detector. The Column was a DB5-MS 30 m × 0.25 mm, 0.25 µm film thickness and the following settings were applied: carrier gas, helium; head pressure, 15 psi; injection volume, 1 µL; injector temperature, 220 °C; spitless injection, 1 min split delay; detector transfer line, 300 °C; source, 70 keV. Temperature program: 50 °C for 1 min, program at 4 °C/min to 220 °C, program at 20 °C/min to 250 °C and hold at 250 °C for 10 min.

The same method was applied for the HS-SDME reaction mix. This method was applied in triplicate for all extracts.

For the separation of total carbonyl compounds obtained by solid-phase reagent extraction, a modified Girard T derivatizing reagent was selected, i.e., the 4-hydrazinyl-*N*, *N*, *N*-trimethyl-4-oxobutan-1-aminium iodide (HTMOB) proposed by Johnson (2007) [24] to improve the sensitivity of high-mass aldehydes and ketones analysis, this reagent is not commercially available and so, we synthesized HTMOB in the lab.

For identifying the aldehydes in our extracts, we first developed a database of mass spectra based on the standard aldehydes we derivatized with HTMOB. A series of aldehyde standards was chosen based on bibliographic works reporting the presence of aldehydes in food [25], then we compared it with our extract. For that we prepared the following solutions: HTMOB 50 mM in MeOH containing 1% acid formic, reference aldehydes 50 mM in MeOH, meat fond was filtered with a filter of 0.45 µm and purified by partitioning with tert-n-butyl methyl ether, finally, a dilution of 100 times with MeOH (HPLC grace) of the extract by condensation with the silica-gel supported reagent. 

In Eppendorf tubes, 100 μL of the solution of HTMOB were added to 100 μL of each standard aldehyde, incubated 10 min at room temperature, brought to 1 mL with methanol and infused in the mass spectrometer. For extracts and cooking juices, 20 µL of HTMOB 1 mM was added to 10 µL of samples; the reaction mix was filtered on a 0.45 µm Teflon filter before injection.

The Micromass Quattro Premier (Waters), driven by the MassLynx 4.0 software, was used in direct injection with the following settings: capillary voltage, 3.1 kV, 80 °C; cone voltage, 20 V, 120 °C; extractor voltage, 4V; analysis flow, 10 µL/min. ESI spectrum were recorded in positive mode, applying full scan, daughter scan, and neutral loss.

### 4.4. Identification of Artefactual Compounds Likely to Form by Reaction with Endogenous Amines

The method to identify such an “artifactual” formation of β-carboline alkaloids or isoquinoline was deduced from Bunel, et al. [63]. It is based on direct contact in equimolar concentrations between the aldehydes identified in the meat extracts and neurotransmitter for 16 h at 37 °C, in aqueous medium; when the aldehyde or neurotransmitter was not soluble in water, methanol was our second choice, whereas, to solubilize tryptophan, an alkaline medium was used. 

TLC analysis was performed after reaction on TLC Silica gel 60 F254, 10 × 20 cm, Merck, Germany, 5 μL of standard and sample were applied with Camag^®^ Automatic TLC Sampler 4, at laboratory temperature. The plates were eluted in automatic developing chamber ADC2 (mobile phase, ethyl acetate—acetic acid—methanol (6:2:2, *v/v*)), observed under UV366 nm and 254 nm in a TLC visualizer 2, and sprayed with ninhydrin reagent; then they were heated at 105 °C for 5 min with a derivatizer and TLC plate heater III of Camag^®^. 

A reaction was confirmed by the presence of at least one spot that did not match any spot in both reactant molecules.

### 4.5. Identification of Alkaloid Artifacts by HPTLC/MS

The samples identified for a positive reaction between an aldehyde and biogenic amines were applied on MS grade TLC plate that was migrated in the same conditions; the spots corresponding to reaction products were localized under UV 254 nm. The plate was transferred to the HPTLC/MS interface (Camag^®^), the spots of interest were eluted one by one with 0.5% formic acid in LC-MS grade methanol and transferred to the mass spectrometer inlet for infusion in the mass spectrometer, mass spectra were acquired in positive mode with full-scan mass spectral acquisition. After dialysis/infusion of all spots of interest, the plate was sprayed with ninhydrin to verify the correctness of spot localization.

### 4.6. In-Silico Prediction Evaluation of Neurotoxicity and Neuroprotection

From the various methods generating models to predict toxicity endpoints, we could access e-MolTOx and pkCSM in addition to the database of PubChem.

The selected compounds were entered in the database using their SMILES structure formats.

The in silico study was started with eMolTox web server that was asked for CNS toxic prediction. Then, the in silico prediction of pharmacokinetic properties (ADMET, i.e., absorption, distribution, metabolism, excretion, and toxicity) was obtained from the pkCSM web server for the six identified compounds. Finally, the obtained results were crossed with PubChem information for the same molecule or a nearby molecule.

### 4.7. Evaluation of Neurotoxic or Neuroprotective Effects In Vitro

For the in vitro toxicity tests, we proposed to apply a 3D model, based on spheroids auto-assembled in lab-molded agarose microwells, that might be more relevant than 2D models. The model of Dingle YT et al. (2015) [64] was applied with modification regarding the source of cells (postnatal rat for Dingle et al. vs. prenatal rat or mouse). All animal procedures and experiments were performed in accordance with the guidelines established by the European Communities Council (Directive 2010/63/EU of 4 March 2014) and approved by of UMONS veterinary ethics committee of the Faculty of Medicine and Pharmacy (Approval No: LA2500635T protocol approval GO/04/02).

The evaluation of a compound effect was applied on day 14 of culture; the medium was fully exchanged with a fresh medium containing the test compounds at concentrations of 50, 100, and 250 µM. 

To measure the cell viability, a resazurin assay was performed as described by Pamies et al. 2018 [60]. After 21 h exposure to the different concentrations of test compound, resazurin (50 μL of a 2 mg/mL stock solution in PBS) was directly added to the medium of 24-well plates. After incubation for 3 h at 37 °C, 5% CO_2_, 50 μL of the medium of each well were transferred to a 96-well plate and the fluorescence intensities were measured at 525 nm/580–640 nm with a multi-well fluorometric reader GloMax^®^-Multi+ Microplate Multimode reader (Promega, Inc., Madison, WI, USA).

To measure the reactive oxygen species (ROS), a highly sensitive fluorometric method described by Silveira et al., 2002 [65] was applied on the cultures at day 14, after the addition of tested compounds. After 24 h of exposure to test compounds, 50 µL of the medium was collected, transferred to a 96-wells plate, and incubated with 50 µL of DCFH 10 µM (45 min, 37 °C, 5% CO_2_). The fluorescence intensity was measured at 490 nm/510–570 nm in a multi-well fluorometric reader GloMax^®^-Multi+ Microplate Multimode reader (Promega, Inc., Madison, WI, USA). 

## 5. Conclusions

The present work aims at investigating the eventual implication of dietary compounds in ET. We then developed a methodology for investigating carbonyl compounds produced during the Maillard reaction in overcooked chicken that could be implicated in the generation of biologically active artifactual endogenous alkaloids. Our work was carried out for a major aldehyde identified in overcooked chicken meat (phenylacetaldehyde, the Strecker degradation product of phenylalanine) and its condensation product with dopamine, i.e., the alkaloid 1-benzyl-1,2,3,4-tetrahydroisoquinoline-6,7-diol (1-benz-6,7-diol THIQ). This alkaloid was evaluated in our neurosphere model for cytotoxicity and oxidative stress induction. Our in vitro data do not support an eventual neurotoxic effect for 1-benz-6,7-diol THIQ.

This research clearly illustrates that despite the challenges in 3D culture and data obtaining, this model could allow a fine study of neurodegenerative disorders and the effect of chemicals on the brain.

## Figures and Tables

**Table 1:** Analysis of ESI-MS/MS neutral loss of 59 Da spectrum of the HTMOB- derivatized carbonyl extract obtained from overcooked chicken meat by condensation with silica-gel supported reagent.

*m/z* of Major Peak in *M^+^* Daughter Scan	*m/z* of Fragment Loss	Identity of Parent Compound
159.95		HTMOB
201.87	60.02	Glycolaldehyde
213.82	71.98	Isobutyraldehyde
227.98	86.14	Isovaleraldehyde or 2-methylbutyraldehyde
262.02	120.07	Phenylacetaldehyde

**Table 2:** Analysis of ESI-MS/MS neutral loss of 59 Da spectrum of the HTMOB- derivatized carbonyl extract obtained from analysis browned meat juices sample.

*m/z* of Major Peak in *M^+^* Daughter Scan	*m/z* of Fragment Loss	Identity of Parent Compound
160.19		HTMOB
214.21	72.02	Isobutyraldehyde
228.21	86.06	Isovaleraldehyde or 2-methylbutyraldehyde
186.24	44.09	Acetaldehyde
172.15	29.98	Formaldehyde

**Table 3:** Identification of carbonyl compounds in browned chicken meat juices extracted by SAFE and derivatized with PFBHA based on RT, KRI, and fragmentation.

*Retention Time T (min)*	*Analytes*	*KRI*	*% Probability (NIST)*
14.677	Propionaldehyde oxime (E)	1088.552	15.90%
14.890	Propionaldehyde oxime (Z)	1094.225	/
16.070	Isobutyraldehyde oxime	1030.819	47.00%
17.631	Butyraldehyde oxime	1077.201	/
19.464	Crotonaldehyde oxime (E)	1033.059	/
20.530	Valeraldehyde oxime (E)	1066.106	/
23.463	Hexanal oxime (E)	951.984	13.20%
23.626	Hexanal oxime (Z)	958.171	2.81%
25.572	Cyclohexanone oxime	1028.913	/
26.277	Heptanal oxime (E)	1053.219	/
26.374	Heptanal oxime (Z)	1056.513	/
29.694	Benzaldehyde oxime	1324.239	62.90%

**Table 4:** Identification of carbonyl compounds in overcooked chicken meat aqueous extract sample extracted by SAFE and derivatized with PFBHA based on RT, KRI, and fragmentation.

Retention Time T (min)	Analytes	KRI	% Probability (NIST)
14.690	Propionaldehyde oxime (E)	1088.901	67.70%
14.890	Propionaldehyde oxime (Z)	1094.225	43.60%
16.009	Isobutyraldehyde oxime	1028.916	51.20%
18.760	Butyraldehyde oxime	1010.226	32.20%
18.905	Butyraldehyde oxime	1014.998	38.70%
19.196	Butyraldehyde oxime	1024.466	39.60%
19.551	Crotonaldehyde oxime	1035.823	0.30%
21.452	3-Methylpentanal oxime	1093.333	5.96%
22.079	4-Methylpentanal oxime	1013.617	21.00%
23.540	Hexanal oxime (Z)	1061.948	0.03%
26.277	Heptanal oxime	1053.219	/
29.694	Benzaldehyde oxime	1073.020	6.40%
31.672	Nonanal oxime	1046.457	/

**Table 5:** Identification of carbonyl compounds in browned chicken meat juices extracted by HS-SDME on PFBHA droplet, based on RT, KRI, and fragmentation.

Retention Time T (min)	Analytes	KRI	% Probability (NIST)
14.676	Propionaldehyde oxime (E)	1088.525	63.70%
14.889	Propionaldehyde oxime (E)	1094.199	65.50%
16.009	Isobutyraldehyde oxime	1028.916	53.20%
18.767	Pentanal (Valeraldehyde) oxime	1010.458	52.30%
18.899	Valeraldehyde oxime	1014.802	20.60%
19.190	Isovaleraldehyde oxime	1024.272	24.50%
19.440	Crotonaldehyde oxime	1032.294	/
22.080	Glycolaldehyde oxime!	1013.651	2.70%
22.246	Glycolaldehyde oxime!	1019.301	10.80%
23.470	Hexanal oxime (E)	1059.701	/
23.540	Cyclopentanone oxime	954.912	80.70%
26.276	Heptanal oxime (E)	1053.185	/
26.860	5-Methoxy-2-pentanone oxime	1072.832	40.60%
29.680	Benzaldehyde oxime	1072.528	72.90%
31.000	Glycolaldehyde oxime or Phenylacetaldehyde oxime	1020.685	14.9%, 5.8%

**Table 6:** Identification of carbonyl compounds in overcooked chicken meat aqueous extract extracted by HS-SDME on PFBHA droplet, based on RT, KRI, and fragmentation.

Retention Time T (min)	Analytes	KRI	% Probability (NIST)
14.676	Propionaldehyde oxime (E)	1088.525	63.70%
14.889	Propionaldehyde oxime (E)	1094.199	65.50%
16.009	Isobutyraldehyde oxime	1028.916	53.20%
18.767	Pentanal (Valeraldehyde) oxime	1010.458	52.30%
18.899	Valeraldehyde oxime	1014.802	20.60%
19.190	Isovaleraldehyde oxime	1024.272	24.50%
19.440	Crotonaldehyde oxime	1032.294	/
22.080	Glycolaldehyde oxime!	1013.651	2.70%
22.246	Glycolaldehyde oxime!	1019.301	10.80%
23.470	Hexanal oxime (E)	1059.701	/
23.540	Cyclopentanone oxime	954.912	80.70%
26.276	Heptanal oxime (E)	1053.185	/
26.860	5-Methoxy-2-pentanone oxime	1072.832	40.60%
29.680	Benzaldehyde oxime	1072.528	72.90%
31.000	Glycolaldehyde oxime or Phenylacetaldehyde oxime	1020.685	14.9%, 5.8%

**Table 7:** Summary of identified condensation reaction between biogenic amines and aldehydes references identified in SAFE and HS-SDME extraction.

	Dopamine	Serotonin	GABA	Nor-epinephrine	Tryptamine	Tryptophan
Glycolaldehyde	+	+	−	−	+	−
2-methylbutyraldehyde	−	−	−	−	+	−
Isovaleraldehyde	−	−	−	−	−	+
Phenylacetaldehyde	+	+	−	−	+	−
Isobutyraldehyde	−	−	−	−	−	−
Benzaldehyde	−	−	−	−	+	−
Hexanal	+	+	−	−	+	−
Propionaldehyde	-	-	−	−	−	−

## Data Availability

Not applicable.
